# The potential of a new larviciding method for the control of malaria vectors

**DOI:** 10.1186/1475-2875-9-142

**Published:** 2010-05-25

**Authors:** Gregor J Devine, Gerry F Killeen

**Affiliations:** 1 Ifakara Health Institute, Dar es Salaam, United Republic of Tanzania; 2 Liverpool School of Tropical Medicine, Liverpool, UK

## Abstract

Malaria pathogens are transmitted to humans by the bite of female Anopheles mosquitoes. The juvenile stages of these mosquitoes develop in a variety of water bodies and are key targets for vector control campaigns involving the application of larvicides. The effective operational implementation of these campaigns is difficult, time consuming, and expensive. New evidence however, suggests that adult mosquitoes can be co-opted into disseminating larvicides in a far more targeted and efficient manner than can be achieved using conventional methods.

## Introduction

The basic tools for malaria vector control are the insecticide-treated bed net (ITN) and indoor residual spraying (IRS). These have a considerable impact on malaria transmission [1,2] by exposing female, host-seeking mosquitoes to insecticide-treated surfaces every time they enter a house to take a blood meal. Repeated contacts over the life cycle amplify the impact of these tools on transmission, even though their effect on mosquito density may remain limited [3]. However, fundamental limitations regarding the coverage of houses or sleeping spaces [3,4] ensure that ITNs and IRS alone may not stop malaria transmission in intensely endemic regions [5]. Moreover, these tools will not be optimally effective in areas where mosquitoes exhibit outdoor resting and biting behaviours, or where the widespread use of ITNs and IRS has controlled endophillic mosquitoes, but left a smaller, more intractable population of exophillic and exophagic mosquitoes behind (e.g. the appearance of *Anopheles arabiensis *as the most abundant vector in areas once dominated by *An. gambiae *and *An. funestus *[6,7]). The sustainability of IRS and ITNs is further threatened by the appearance of pyrethroid resistance in some mosquito populations [8]. All of these factors require that novel but complementary control methods are developed, that use novel insecticide classes that are not yet resisted.

Targeting the aquatic habitat is one obvious additional strategy. This is an increasingly valued approach in many African settings [9] but larvicides, unlike IRS and ITNs, act on a single, non-transmitting stage in the mosquito lifecycle and can only impact disease by reducing vector abundance [10]. The myriad and cryptic nature of aquatic habitats and the difficulty in identifying and targeting the most productive of these [10] makes maximizing that impact very challenging.

## An efficient new larviciding technique

A recent field trial with the dengue vector, *Aedes aegypti*, exploited the obligate behaviours of adult mosquitoes to transfer a potent larvicide between resting and oviposition sites [11]. An impressive impact on the juvenile population was mediated by 1) a highly effective and persistent insecticide, 2) the predictability of sites where adult mosquitoes could be exposed, 3) a limited aquatic habitat and 4) sufficient mosquito density. The use of adult females as larvicide-disseminating vehicles resulted in the very precise targeting of the insecticide; only those aquatic habitats visited by adults were contaminated and, the more popular the site, the greater the number of transfer events. Treatment of a small proportion of resting places (≤5%) resulted in high coverage of aquatic sites (>95%). This amplification was facilitated by an abundance of mosquitoes, the potential for multiple resting-oviposition cycles (i.e. contamination events) over a single mosquito lifetime and the persistence and potency of the insecticide. Pyriproxyfen (PPF) has no discernable effects on adult longevity or behaviour, but renders larval habitats unproductive for long periods at tiny concentrations [11,12]. It is approved by the World Health Organization (WHO) and has a recommended drinking water limit of 300 ppb, which is orders of magnitude above the concentrations required for mosquito control [12]. It is unrelated to any other WHO approved adulticide or larvicide and resistance has not yet been documented in any mosquito species.

## Potential impact on malaria vectors

The auto-dissemination of PPF is well suited to the localized and predictable resting and dispersal habits of *Ae. aegypti *[11]. That species also exhibits prolonged contact with the aquatic habitat during oviposition [13] which serves to maximize the transfer of PPF between resting and oviposition sites. Anopheline adults have more variable dispersal patterns [14] and are variously described as laying eggs whilst settled on the water, perched above the water or whilst hovering over the surface [13,15]. Definitive field studies are lacking, but the propensity of the female to contact the water during oviposition will clearly affect the efficacy of PPF transfer.

Deterministic simulation modelling can further help to consider how the auto-dissemination of PPF might impact Anopheline mosquitoes. The model is described elsewhere [11] and describes the relationship between the effective coverage of adult resting sites (C_r_) and larval habitats (C_h_) with PPF using a simple exponential function of the time over which contaminated habitats remain unproductive (U), the number of ovipositions (O) by the adult population, the number of larval habitats (H), and the number of contaminating events needed to make a single habitat unproductive (Ω):(1)

**Figure 1:**
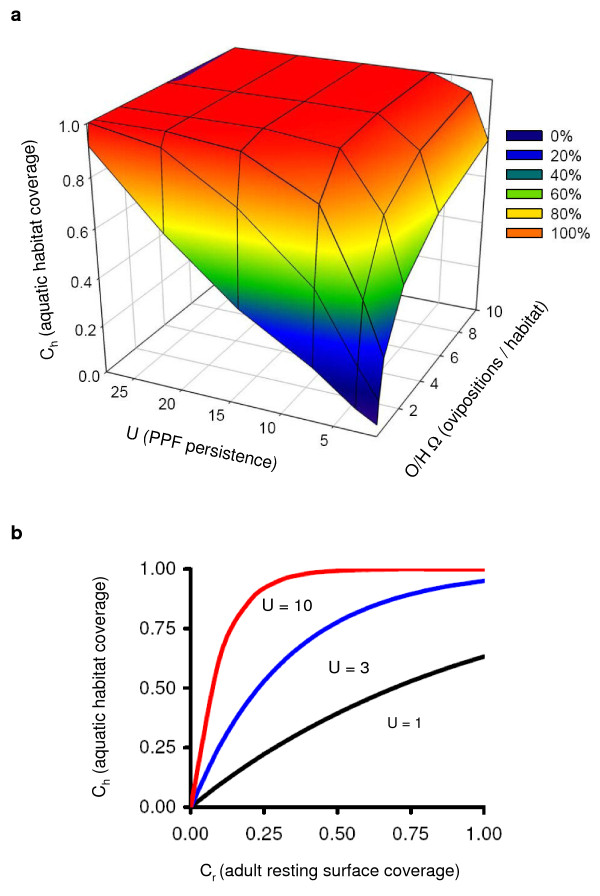
**Deterministic simulation model outcomes**. a) Resting site coverage (C_r _= 0.5) can be amplified by insecticide persistence (U) and the number of contamination events per habitat (O/H) to achieve complete coverage of the aquatic habitat (C_h_). b) Under stable conditions of contamination (O/H Ω = 1) the persistence of the insecticide and (by implication) the stability of the habitat (U) is of key importance to this amplification.

Some key malaria vectors in sub-Saharan Africa, such as *An. gambiae *s.s. and *An. funestus*, feed predominantly upon humans in and around houses [16]. Their specialized behaviours make them devastating carriers of malaria, but ideal targets for vector control. They are so adapted to humans that the use of the ITNs and IRS to target their obligate host-seeking and indoor resting behaviours can displace them from wide areas [7,17]. Treatment of similar barrier and resting surfaces with PPF might therefore result in a high proportion of contact sites being contaminated (C_r _≥ 0.5). It is speculated that almost total coverage of the juvenile habitat (C_h _≥ 0.99) can be achieved if breeding sites (H) are stable enough to ensure the persistence of the larvicide (U ≥ 14) and if a favourable ratio of ovipositing mosquitoes to aquatic sites exists (O/H ≥ 1). This is illustrated in Figure 1a. The importance of the larvicide's persistence (and by implication, the stability of the habitat) is emphasized in Figure 1b.

For *An. gambiae *s.l., the requisite of a favourable O/H ratio is least likely to occur when oviposition sites are constantly being created or flushed by rainwater or are so large that they require an unfeasibly high number of contaminating events (Ω) given a limited density of adult mosquitoes and an abundance of aquatic habitats (O/H). However, in many sub-Saharan regions, dry seasons reduce flushing effects and render breeding sites less common. These stable, limited habitats are essential for mosquito survival [18] and are suitable for cumulative contaminations by adult mosquitoes. Dry-season control is often central to the success of habitat management strategies for *An. gambiae *[18-20].

An auto-dissemination strategy using PPF might be most useful against vectors that are not readily managed by ITNs or IRS. *An. arabiensis *is an important malaria vector that feeds and rests on and near livestock [21]. It is therefore less vulnerable to the insecticide treatment of indoor surfaces than *An. gambiae *s.s. or *An. funestus*. In some areas, it is an increasingly important focus of control campaigns now that these latter species are being successfully displaced. It is possible that high coverage of the resting and feeding sites of *An. arabiensis *with PPF might be achieved by treating cattle and/or animal corrals (C_r _> 0.5). The recent development of some highly effective baits for Anopheline mosquitoes [22,23] could also be exploited to lure and contaminate large numbers of female mosquitoes. The advantage of this "lure and disseminate" technique over a simple "lure and kill" approach lies in the potential amplification of coverage at the aquatic habitat (C_h_) and in the utilization of a novel chemistry for which resistance has not yet been documented.

*An. funestus *is an example of an important malaria vector that may be less sensitive to the auto-dissemination technique. Its larvae tend to be restricted to larger aquatic sites that are regularly flushed with water [16]. This will decrease the persistence of PPF (U) and increase the number of contaminations necessary to render habitats unproductive (Ω). However, under some conditions, the relatively stable demographic composition of *An. funestus *[16] might contribute to maintaining a practicable O/H ratio.

Optimizing the contamination of aquatic sites, particularly in the context of large water volumes or low density mosquito populations, might be possible through increasing the potency of the PPF formulation (such that Ω is minimized and U is maximized). The levels of control detailed by Devine *et al *[11] were achieved with a commercially available 0.5% PPF formulation. Increasing the percentage active ingredient to 50% would raise the contaminating potential of a single transfer event by 100-fold. Additional ecological factors might further maximize the number of contaminating transfers between treated surfaces and aquatic habitats. Single breeding sites often receive visits from a number of Anophelines and repeated sequences of resting and oviposition may occur during a single gonotrophic cycle [24]. Perhaps crucially, many abundant, non-target mosquitoes may also be enlisted in the transfer process; Culex and Anopheline species often share the same aquatic and terrestrial resources [18]). Yet another potentially exciting element of the strategy, still to be explored in a field setting is that contact with PPF reduces the fertility of mosquitoes [12]. This trait has been exploited for the control of other dipteran pests [25].

## Conclusion: an exciting new tool?

The obligate resting and oviposition behaviours exploited by this novel technique underpin mosquito survival, reproduction and abundance but they have been largely overlooked in deference to studies on host-seeking and blood-feeding. Although the role of conventionally applied larvicides for the control of malaria vectors has been convincingly demonstrated [9,19] the optimization and application of this new auto-dissemination methodology will require a detailed characterization of oviposition behaviour and of the effective transfer distances between feeding, resting and aquatic resources. It will also require an understanding of the abundance and behaviour of co-existing species at those resources and a study on the impacts of PPF on various aquatic habitats. In principle however, the method offers a new way to reduce mosquito densities (and therefore affect entomological inoculation and malaria rates [10]). Its concepts are elegantly simple and safe and it promises substantial reductions in the financial and labour costs of larviciding. It may ultimately prove to be a useful complement to ITNs and IRS, especially in areas where these are threatened by the evolution of pyrethroid resistance and where mosquitoes rest and bite out of doors.

## Competing interests

The authors declare that they have no competing interests.

## Authors' contributions

GD wrote the article, GK devised the model. All authors read and approved the final manuscript.
